# Genetic and plastic rewiring of food webs under climate change

**DOI:** 10.1111/1365-2656.13541

**Published:** 2021-06-22

**Authors:** Matthew A. Barbour, Jean P. Gibert

**Affiliations:** ^1^ Department of Evolutionary Biology and Environmental Studies University of Zurich Zurich Switzerland; ^2^ Department of Biology Duke University Durham NC USA

**Keywords:** eco‐evolutionary dynamics, ecological networks, food webs, genotype‐by‐environment interactions, phenotypic plasticity, quantitative genetics

## Abstract

1. Climate change is altering ecological and evolutionary processes across biological scales. These simultaneous effects of climate change pose a major challenge for predicting the future state of populations, communities and ecosystems. This challenge is further exacerbated by the current lack of integration of research focused on these different scales.
2. We propose that integrating the fields of quantitative genetics and food web ecology will reveal new insights on how climate change may reorganize biodiversity across levels of organization. This is because quantitative genetics links the genotypes of individuals to population‐level phenotypic variation due to genetic (G), environmental (E) and gene‐by‐environment (G × E) factors. Food web ecology, on the other hand, links population‐level phenotypes to the structure and dynamics of communities and ecosystems.
3. We synthesize data and theory across these fields and find evidence that genetic (G) and plastic (E and G × E) phenotypic variation within populations will change in magnitude under new climates in predictable ways. We then show how changes in these sources of phenotypic variation can rewire food webs by altering the number and strength of species interactions, with consequences for ecosystem resilience. We also find evidence suggesting there are predictable asymmetries in genetic and plastic trait variation across trophic levels, which set the pace for phenotypic change and food web responses to climate change. Advances in genomics now make it possible to partition G, E and G × E phenotypic variation in natural populations, allowing tests of the hypotheses we propose.
4. By synthesizing advances in quantitative genetics and food web ecology, we provide testable predictions for how the structure and dynamics of biodiversity will respond to climate change.

## Graphical abstract

The authors synthesize advances in quantitative genetics and food web ecology to understand how climate change will reshape biodiversity across levels of organization. They argue that genetic and plastic phenotypic responses to climate change will rewire food webs in predictable ways, with consequences for ecosystem resilience and eco‐evolutionary dynamics.

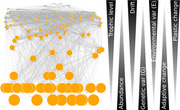

## 1 INTRODUCTION

Climate change is affecting ecological processes across levels of biological organization (Scheffers et al., 2016). Indeed, rapidly rising global temperatures increase the basal metabolic rate of individuals (Brown et al., 2004; DeLong et al., 2018; Gillooly et al., 2001), which has myriad ecological consequences, from changes in the strength and outcome of ecological interactions (Betini et al., 2019; Bideault et al., 2019; Koltz et al., 2018; O'Connor, 2009; Schaum et al., 2017), to shifts in species distributions and abundances (Bonebrake et al., 2018; Freeman & Class Freeman, 2014; Freeman et al., 2018). Yet, accurately predicting the state of most populations, communities and ecosystems in future climates remains challenging (Gaüzère et al., 2018). On the one hand, this is due to direct impacts of climate change on focal species often having cascading (indirect) effects on multiple other populations through networks of shared ecological interactions (Bascompte et al., 2019; Farrer et al., 2015; Gibert, 2019; Van de Velde et al., 2017). But making predictions is difficult also because the adaptive landscape species experience is likely to change as environmental conditions shift (Exposito‐Alonso et al., 2019; MacColl, 2011; Siepielski et al., 2017), thus reshaping the eco‐evolutionary feedbacks that ultimately determine ecological interactions (McPeek, 2017; Raimundo et al., 2018). While we have some understanding as to how specific pairwise interactions will respond to climate change (DeLong & Lyon, 2020; Rall et al., 2010; Vucic‐Pestic et al., 2011), we currently lack a framework for predicting the ecological and evolutionary consequences of climate change for networks of interacting species.

Phenotypes provide the fundamental link between ecological and evolutionary processes within ecological networks in general, and food webs in particular. Ecologically, phenotypes govern population‐level processes like per‐capita birth and death rates (Arendt, 2011; de Roos et al., 2003; Savage et al., 2004), they mediate the strength and organization of species interactions within food webs (Brose et al., 2006; Emmerson & Raffaelli, 2004; Petchey et al., 2008) and determine how species respond to their abiotic environment (Lavorel & Garnier, 2002; Suding et al., 2008). Evolutionarily, heritable phenotypes that influence fitness are subject to natural selection and evolutionary change (Fisher, 1930; Lande, 1976). Phenotypic evolution can therefore affect multiple ecological processes, whereas ecological dynamics and species interactions can, in turn, mediate the pace and direction of phenotypic change (Abrams, 1991; Abrams & Matsuda, 1997; Hairston et al., 2005; Schaffner et al., 2019; Van Velzen & Gaedke, 2017). Although how slow species diversification determines food web structure has been addressed to some extent (Allhoff et al. 2015; Brännström et al., 2011; Ingram et al., 2009; Loeuille & Loreau, 2005), the role of rapid evolution in shaping the structure and dynamics of food webs, and how the structure of these networks, in turn, constrain or promote phenotypic change are less well understood (Barbour, Greyson‐Gaito, et al., 2020; Gibert & Yeakel, 2019; Guimarães Jr et al., 2017; McPeek, 2017; Romanuk et al., 2019). Additionally, how these eco‐evolutionary feedbacks can mediate food web responses to climate change is largely unknown, as studies often neglect the network context that species are embedded in (but see Moya‐Laraño et al., 2012; Yacine et al., 2021).

Given the fundamental importance of phenotypic variation for ecological interactions, we argue that integrating quantitative genetics and food web theory will enable us to better understand and predict the consequences of climate change across biological scales. Quantitative genetics links the genotypes of individuals to population‐level phenotypic variation of complex traits (Falconer & Mckay, 1996). Quantitative genetics posits that standing phenotypic variation (P) can be partitioned into genetic and plastic sources of variation. Genetic variation results from an organism's genotype and can itself be partitioned into additive (sum of the effects of one or more loci on a phenotype), dominance (interactions between alleles at the same loci) and epistatic genetic effects (interactions between alleles at different loci). Here, we focus on additive genetic effects (G hereafter), as it is this source of variance that determines the response to selection (Falconer & Mckay, 1996; Fisher, 1930; Lush, 1937) and is also the greatest contributor to the total genetic variance of complex traits (Hill et al., 2008). Phenotypic plasticity occurs when there is a change in phenotypic expression of a genotype in response to the environment and can be partitioned into average responses among genotypes (E) and genotype‐by‐environment interactions (G × E), which occur whenever genotypes vary in their response to the environment (Bradshaw, 1965; Scheiner & Goodnight, 1984). This later source of variation is often ignored and the one we know the least about in the context of climate change and ecological networks. Although not without its challenges, it is now possible to quantify these sources of phenotypic variation using modern genomic and statistical tools, even in non‐model organisms, under natural conditions (Box 1). Quantitative genetics thus link phenotypes to their environment in a disarmingly simple way, and phenotypes bridge ecology and evolution. We thus propose that integrating quantitative genetics and food web theory provides a lens through which to understand and predict how shifts in environmental conditions may affect food webs, and how food web structure may, in turn, mediate these effects down to the genetic level.

BOX 1 **Partitioning G, E and G × E in the age of genomics**

Traditionally, common garden experiments across multiple environments have been used to partition G, E and G × E in phenotypic traits and associated species interactions (Barbour et al., 2019; Cooper et al., 2019; Faticov et al., 2019). Because of this, most prior work has been biased towards quantifying G, E and G × E effects of plants on their associated communities. However, recent advances in genomics have now made it possible to partition trait variation due to G, E and G × E in non‐model organisms in natural populations. Tools such as restriction‐associated DNA sequencing (RAD‐seq; Baird et al., 2008), multiplexed shotgun genotyping (Andolfatto et al., 2011) and genotype‐by‐sequencing (GBS; Elshire et al., 2011) permit thousands of single nucleotide polymorphisms (SNPs) to be collected across the genome. These genetic markers can then be used to estimate a genetic relationship matrix (GRM) that, when combined with environmental factors and individual trait (or interaction) data, can be used to statistically partition sources of G, E and G × E (Kerin & Marchini, 2020; Yang et al., 2011). Until recently, these statistical tools have primarily been used to derive genomic‐based estimates of G and narrow‐sense heritability (Yang et al., 2011). Note that including relevant environmental factors is critical for obtaining accurate estimates of heritability, but beyond that, these sources of variation are often viewed as a nuisance. Instead, this paper emphasizes that E and G × E are interesting sources of phenotypic variance, as they give us a more complete picture of how organisms will respond to changes in environmental conditions associated with climate change.

Although advances in genomic and statistical tools now permit the analysis of G, E and G × E, there are at least two important points to keep in mind when planning a project that will use genomic data to partition sources of phenotypic variation (Stanton‐Geddes et al., 2013). The first is that tens of thousands of SNPs are necessary to provide accurate estimates of G and narrow‐sense heritability (Stanton‐Geddes et al., 2013). This has become less of a limitation, especially with recent advances in reduced‐representation genotyping (Andolfatto et al., 2011; Baird et al., 2008; Elshire et al., 2011). The second is that hundreds of individuals (or genotypes) are necessary to provide accurate estimates of heritability (Stanton‐Geddes et al., 2013). We see this second point as the current rate‐limiting step in the broad‐scale application of these genomic approaches to natural populations. This is even more of a concern for getting precise estimates of G × E, if we consider the general rule that more data are needed for precise estimates of statistical interactions. While we do not want to discourage researchers from studying G × E, as not accounting for this source of variance can inflate estimates of E (Visscher et al., 2008) and underestimate heritability in future climates, it is important to bear in mind that the uncertainty in G × E will be inherently higher than for G or E.

Recent eco‐evolutionary models have begun to bridge quantitative genetics and food web ecology to understand the feedback between phenotypic evolution and network structure (Gibert & Yeakel, 2019; Guimarães Jr et al., 2017; McPeek, 2017). Classic models that track the dynamics of species interactions within food webs can be re‐expressed in terms of per‐capita growth rates, or the natural logarithm of mean absolute fitness of individuals in a population (Abrams et al., 1993; Lande, 1976). As a consequence, these eco‐evolutionary models can also track how changes in mean fitness—and concomitant phenotypic change—affect population and community dynamics (e.g. Cortez & Ellner, 2010; de Andreazzi et al., 2018; McPeek, 2017; Schreiber et al., 2011). While these models routinely account for genetic change in mean phenotypes through G in the form of narrow‐sense heritability (*h* ^2^ = G/P), they implicitly assume that the underlying components of phenotypic variation (G, E and G × E) remain constant over time and do not change with environmental conditions, as they do in nature. However, if any of the underlying components of phenotypic variation change with the environment, so will *h* ^2^, which may, in turn, influence phenotypic evolution and ecological dynamics.

On the other hand, most ecological models that account for changes in environmental conditions implicitly incorporate E in the form of plastic changes in demographic rates and interaction strengths with changes in an underlying environmental variable like temperature (e.g. Bernhardt et al., 2018; Binzer et al., 2016; Dell et al., 2014; Osmond et al., 2017; Vasseur et al., 2014). This is typically done without acknowledging that the modelled effect of temperature is likely plastic. However, this approach does not incorporate changes in genetic variation (G) in response to environmental change nor genetic variation in plasticity (G × E). Bridging these two distinct modelling approaches, conceptually and mathematically, is key to understanding how populations within food webs respond to climate change.

Here, we synthesize how genetic (G) and plastic (E and G × E) responses to climate change may affect the phenotypes that ultimately mediate ecological interactions within food webs. While previous work has focused on changes in mean phenotypes, we emphasize the role of changes in phenotypic variance for food web structure and dynamics, because we expect these effects to be more predictable in the context of climate change. We also propose ways to account for G, E and G × E in eco‐evolutionary models to shed new light on how climate change may affect food webs. Finally, we discuss how the structural role a species may play within a food web may promote or constrain eco‐evolutionary dynamics and how these effects may, in turn, mediate the impacts of climate change. Throughout, we argue that by understanding how changes in environmental conditions may affect different sources of phenotypic variation, we can surmise how food webs may be genetically or plastically rewired as the climate changes. Although our focus here is on food webs, we expect that many of these predictions may apply to other ecological networks as well.

## 2 QUANTITATIVE GENETICS AND THE PACE OF PHENOTYPIC CHANGE

### 2.1 Mean and variance

Climate variation can influence the fitness landscape (i.e. the relationship between genotypes/phenotypes and reproductive success) and natural selection acting on traits (Exposito‐Alonso et al., 2019; Merilä & Hendry, 2014; Siepielski et al., 2017). As a consequence, environmental change can lead to adaptive evolution in the mean and variance of phenotypes that mediate ecological interactions (Barbour, Greyson‐Gaito, et al., 2020; Bolnick et al., 2011; Dehling et al., 2016; Emmerson & Raffaelli, 2004; Gibert et al., 2015; Hart et al., 2016). While evolutionary change in mean phenotypes is always accounted for in eco‐evolutionary models (Jones et al., 2009; Schreiber et al., 2011; Vasseur et al., 2011), changes in phenotypic variation typically are not (but see Nuismer et al., 2005; Taper & Chase, 1985 for evolutionary models that allow variances to evolve, and DeLong & Gibert, 2016; Melián et al., 2018 for eco‐evolutionary models that do so). Yet, changes in both mean and variance will have important consequences for ecological interactions (Bolnick et al., 2011; Gibert & Brassil, 2014; Gibert et al., 2015; Gibert & DeLong, 2015; Hughes et al., 2008, 2015; Violle et al., 2012) and will determine the ability of species to respond adaptively and plastically to environmental change (Hunter et al., 2018; Steele et al., 2011). So, in addition to studying changes in mean phenotypes, partitioning the contribution of G, E and G × E is key to developing a mechanistic understanding of how ecological interactions will respond to climate change. In what follows, we first focus on how changes in the different sources of phenotypic variation affect food webs (Sections 3 and 4) and we then focus on how changes in mean phenotypes influence food webs (Section 5).

### 2.2 Pace and time‐scale

Changes in genetic variation (G) are likely to lag behind environmental change, as evolutionary change is an inter‐generational process (Fox et al., 2019). Plasticity (E and G × E), on the other hand, can operate within generations so that environmental change directly affects phenotypic variance at a temporal scale much closer to that at which the environment is changing, leading to faster phenotypic responses that may or may not be adaptive (Chevin et al., 2010; Fox et al., 2019). Interestingly, certain forms of G × E can alter the expression of G within generations (Wood & Brodie III, 2016), effectively shifting the time‐scale of future trait change by altering narrow‐sense heritability (*h* ^2^ = G/P). Of course, rapid evolution can and does happen (DeLong et al., 2016; Hairston et al., 2005; Hendry, 2016; McPeek, 2017; Schaffner et al., 2019; Yoshida et al., 2003), which may also reduce the difference in time‐scales (Bassar et al., 2021).

These differences in time‐scale often have important ecological implications. For example, lags in consumer–resource interactions are a potent source of instability (May, 1973; Murdoch et al., 1987). The longer time‐scale of evolution (change in G) may thus lead to ecological instability, while the shorter time‐scales of plastic change (change in E and G × E) may stabilize ecological dynamics, regardless of whether it is adaptive or not. However, rapid phenotypic responses can be both stabilizing and destabilizing (Abrams, 1992; Cortez, 2016). For example, whenever phenotypes evolve rapidly, the final direction of the effect ultimately depends on how much G there is in each interacting species, and whether selection reduces or increases G: stabilizing selection is mostly stabilizing (in terms of the ecological dynamics) while disruptive or short‐term directional selection (as in an arms‐race) often is destabilizing (Cortez, 2016, 2018). However, demographic rates and interaction parameters that ultimately determine how species interact within food webs often have a nonlinear influence on stability (Rip & McCann, 2011). As a consequence, whether phenotypic change is stabilizing or destabilizing will also depend on the mean values for the traits that set those demographic rates and parameters.

### 2.3 Mechanisms of change in G

Climate change can influence the evolution (adaptive or not) of standing genetic variation (G) in predictable ways whenever it shifts species distributions. For example, we expect G to decrease in organisms whose geographical ranges are contracting, due to increasing genetic drift (Arenas et al., 2012; Pauls et al., 2013). On the other hand, climate change can result in an increase in G through gene flow in organisms whose populations are becoming more interconnected (Nadeau & Urban, 2019). Unfortunately, not all changes in G will be predictable: while environmental change may lead to strong directional selection in a novel climate (Exposito‐Alonso et al., 2019; Franks et al., 2007; Shipley et al., 2020), how G responds will depend on how the form and magnitude of selection has been affected by the environment. In that case, G can increase, decrease or remain unaffected. Still, in the absence of disruptive selection, G will generally decrease as stabilizing and directional selection generally act to reduce genetic variance (Arnold, 1992). Fortunately, it is possible to model changes in the fitness landscape using tools from quantitative genetics and eco‐evolutionary dynamics (Arnold, 2003; Lande & Arnold, 1983). Moreover, we can use empirical estimates of selection to infer changes in the shape (curvature) of the fitness landscape that ultimately determines G (Barbour, Greyson‐Gaito, et al., 2020), thereby linking empirical and theoretical approaches.

### 2.4 Mechanisms of change in E and G × E

Climate change can also influence plastic trait variation (E and G × E) in predictable ways. Changes in mean environmental conditions often lead to concordant changes in environmental variability (Easterling et al., 2000; Nijsse et al., 2019; Vasseur et al., 2014). For example, as mean global temperatures rise, temperature variability has been increasing steadily (Bathiany et al., 2018). This is also true for changes in precipitation regimes and water availability as increasingly serious droughts followed by wetter‐than‐average years become more prevalent (Naumann et al., 2018). These increasing fluctuations in environmental conditions ultimately lead to short‐ and long‐term plastic responses (Franks et al., 2014; Merilä & Hendry, 2014; Scheepens et al., 2018), which could increase phenotypic plasticity as organisms cope with novel and more variable environmental conditions. For example, plasticity in flowering time and above‐ground biomass increases with increasing environmental fluctuations in *Arabidopsis thaliana* (Scheepens et al., 2018). More generally, animal and plant populations from more variable temperate regions often exhibit more phenotypic plasticity than populations from more stable, tropical regions (Aguilar‐Kirigin & Naya, 2013; Molina‐Montenegro & Naya, 2012; Pintor et al., 2015; Ren et al., 2020).

Environmental change can also influence the expression of phenotypic variation within generations through G × E interactions (Scheiner, 1993; Wood & Brodie III, 2015). For example, while flowering time can change in response to a rapidly shifting climate (Franks & Hoffmann, 2012), it is also under epigenetic control (Amasino & Michaels, 2010; Michaels, 2009), thus representing G × E. This source of plasticity frequently increases in novel environments (Saltz et al., 2018). We thus expect both sources of plastic trait variation (E and G × E) to increase as organisms cope with novel climates.

## 3 GENETIC (G) REWIRING OF FOOD WEBS

**FIGURE 1:**
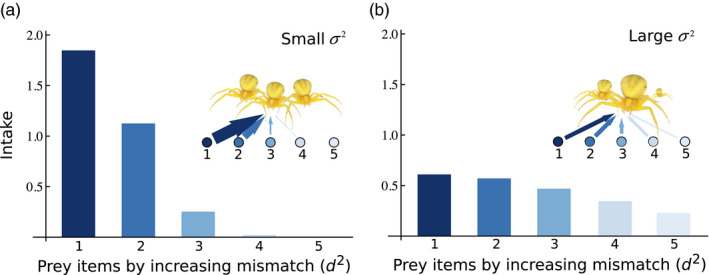
Intake rate for a predator for all species consumed (or potentially consumed) ordered by increasing phenotypic mismatch for increasing levels of phenotypic variation. (a) *σ* ^2^ ~ 0 and (b) *σ* ^2^ = 7. As phenotypic variation increases, the number of interactions increases as well, but their strength weakens. Details of the underlying model are given in Box 2

**FIGURE 2:**
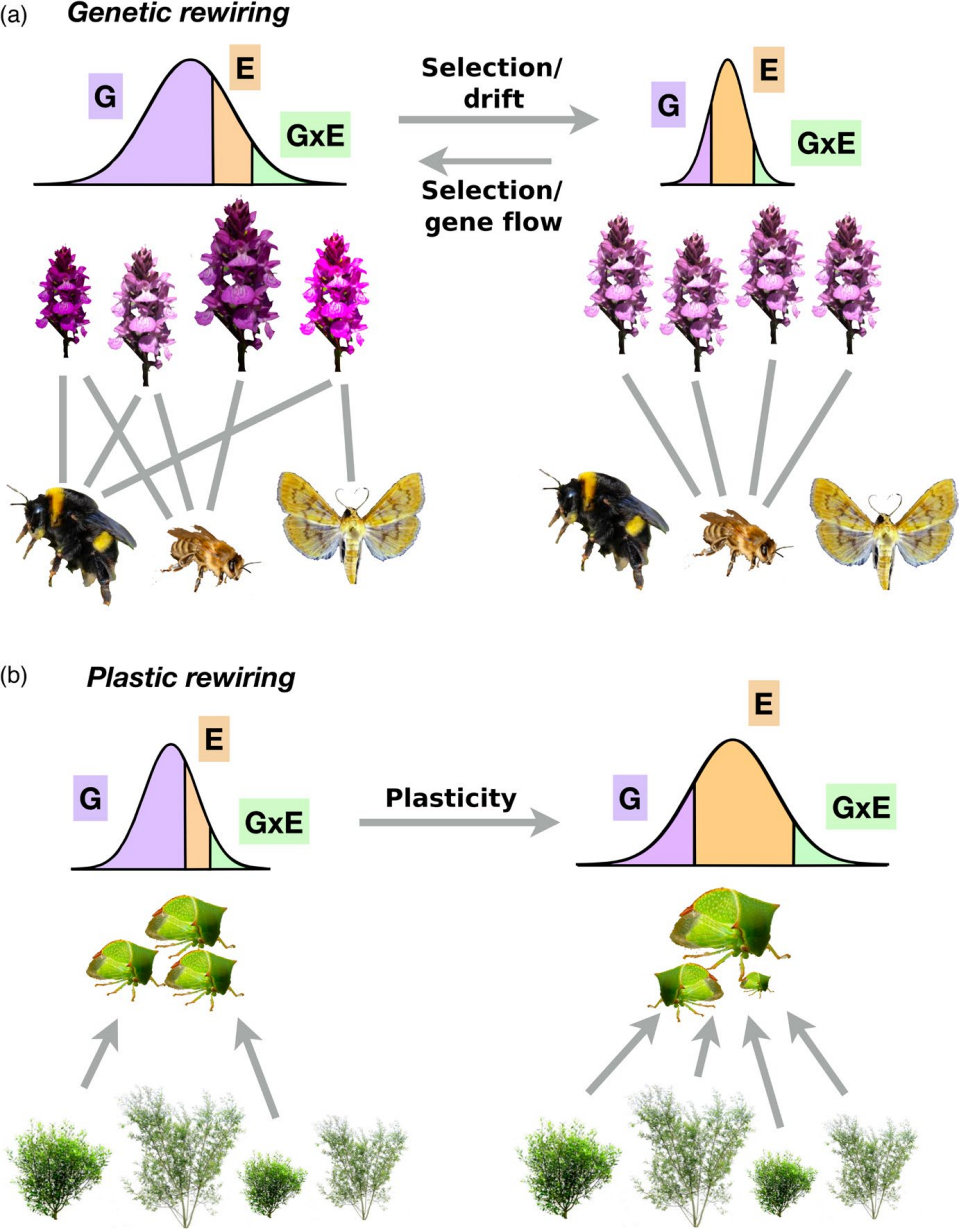
(a) Genetic rewiring occurs whenever genetic variation (G) increases or decreases as the environment changes. Changes in G can be due to changes in the adaptive landscape (selection), range contractions or expansions (drift and gene flow). Changes in G, in turn, lead to predictable rewiring of ecological networks. (b) Plastic rewiring occurs whenever E and G × E change with the environment. Changes in E and G × E are more likely to be unidirectional, given that increasing amounts of environmental variability are expected with climate change. Increases in plasticity, in turn, lead to an increase in the number of interactions in the network. For simplicity, we illustrate a change in E, although G × E also contributes to plastic trait variation

Genetic variation and phenotypic variation determine the number and strength of species interactions within food webs (Barbour et al., 2016; Gibert & DeLong, 2017) (Box 2, Figure 1). When responses to environmental change result in changes in G, we therefore expect the number and strength of ecological interactions to be affected, resulting in predictable *genetic rewiring* of food webs (Figure 2a). Rewiring refers to the reconfiguration of the strength and/or organization of species interactions in a network (Bartley et al., 2019). For example, a decrease in G in a focal plant species (through selection or drift) may lead to phenotypic incompatibility with its pollinators due to reduced phenotypic variability. This would, in turn, result in a smaller number of stronger interactions between plants and the pollinator that consume nectar from them (Figure 2a), also reducing the possibility for indirect ecological and evolutionary effects (Guimarães Jr et al., 2011, 2017). An increase in G, such as that observed in organisms whose populations are becoming more interconnected with climate change, may instead lead to a larger number of weaker interactions (Figure 2a).

There is broad empirical support for genetic variation influencing food web structure. For example, herbivore and pollinator richness increases in genetically diverse populations of tall goldenrod *Solidago altissima* (Crutsinger et al., 2006; Genung et al., 2010), thus increasing the number of interactions in the network. This pattern appears to apply widely in plant–herbivore interactions. For example, increased plant genetic diversity has been linked to reduced damage from generalist herbivores (Koricheva et al., 2018; McArt & Thaler, 2013), therefore suggesting weaker interactions, likely due to increased nutrient variability that generally reduces herbivore performance (Wetzel et al., 2016). Moreover, these genetic effects can indirectly shape food webs at higher trophic levels, generally resulting in a greater number of connections throughout the food web (Barbour et al., 2016). Given that the effects of plant genetic diversity are often as strong as changes in plant species diversity (Koricheva et al., 2018), genetic rewiring likely has important consequences for the architecture of food webs across trophic levels. As both the number and strength of ecological interactions are important drivers of community stability (Allesina & Tang, 2012; May, 1972; McCann, 2000; Neutel et al., 2002; Thébault & Fontaine, 2010), we expect that genetic rewiring will lead to changes in stability (Barbour, Kliebenstein, et al., 2020). The potential for genetic rewiring, therefore, links the genetic makeup of populations to the structure and stability of food webs.

Despite the clear potential for genetic rewiring, we are unaware of any examples that have looked at genetic rewiring resulting from evolved responses to climate change. Populations whose ranges are contracting/expanding or adaptively diverging in response to climate change could be good candidate systems to test our hypotheses, although it would be important to simultaneously consider changes in mean phenotypes (Section 5). In addition, the heritable phenotypes mediating these interactions are often not examined in detail (Crutsinger, 2016; Hughes et al., 2008), in part because multiple traits may simultaneously mediate ecological interactions (Barbour et al., 2015). This makes it difficult to identify which traits to measure in the field. However, genomic tools may provide a new way forward, by revealing cryptic linkages between genes and interacting species (Box 1; Barbour, Kliebenstein, et al., 2020; Barker et al., 2019; Rudman et al., 2018; Sato et al., 2019).

### 3.1 Eco‐evolutionary consequences

Changes in G in response to environmental conditions will alter the outcome and pace of evolution, further influencing food web structure and dynamics (Loeuille, 2019). Because the response to natural selection is proportional to G (Fisher, 1930; Lande, 1976, 1979, 1982), changes in G will alter the ability of populations to adapt to novel environmental conditions (Hansen & Houle, 2008). While theoretical work has shown that changes in the pace of rapid evolutionary dynamics can affect food web structural properties like the number of trophic levels and body size structure (Gibert & Yeakel, 2019), experimental results remain elusive. Ecological outcomes of rapid eco‐evolutionary dynamics can be manifold: rapid evolution may lead to species coexistence when it would otherwise not be possible (Loeuille, 2019; Vasseur et al., 2011). Reductions in G could also lead to a decrease in heritability and eco‐evolutionary dead ends (Carlson et al., 2014), where the partner that is unable to rapidly respond to the ecological interaction may see their abundance greatly reduced (or be extirpated from the system: evolutionary murder, sensu; Loeuille, 2019), thus affecting food web complexity and overall species diversity.

## 4 PLASTIC (E AND G × E) REWIRING OF FOOD WEBS

In the absence of concomitant changes in G, an increase in phenotypic plasticity would lead to increases in the total phenotypic variation of a population and to *plastic rewiring* of food webs (Box 2, Figure 1). Increases in plastic trait variation should lead to a larger number of weaker interactions within food webs and plant–pollinator networks (Box 2, Figure 2b). For example, generalist consumers may rapidly respond to novel environmental heterogeneity created by climate change, which will likely result in a larger number of weaker interactions with their resources (Bartley et al., 2019). In networks where interactions are more specialized, plastic rewiring may be minimal, as the traits mediating specialized interactions are likely under stronger genetic control. For example, different plant secondary chemical compounds often determine resistance to specific herbivore species, and these chemical compounds generally exhibit a high degree of heritability (Barbour et al., 2015; Geber & Griffen, 2003; Johnson et al., 2009). We note that while genetic rewiring could happen in either direction, plastic rewiring is likely to lead to an increase in the total number of interactions, but not to decreases, due to the hypothesized increase in phenotypic plasticity. Because the mechanism through which interactions increase in number also weakens them (Figure 1; Box 2; Gibert & Brassil, 2014; Gibert & DeLong, 2017; Schreiber et al., 2011), plastic rewiring in response to climate change should be mostly stabilizing for food webs (Gellner & McCann, 2012; McCann et al., 1998), thus maintaining species diversity.

BOX 2 **Effects of phenotypic variation on the number and strength of ecological interactions**

Predator–prey interactions are often controlled by a quantitative and normally distributed trait, like body size (Aljetlawi, 2004; Osmond et al., 2017; Vucic‐Pestic et al., 2010). To model such interactions, we assume that foraging rates are maximized at a certain trait value *X*_opt_, which optimizes the parameters controlling the functional response of the predator: attack rate is maximal at *X*_opt_ and the handling time is minimal, while the attack rate decreases and the handling time increases away from that optimum (Barrios‐O'Neill et al., 2016; Gibert & Brassil, 2014). For example, if the predator is much larger, or smaller, than its prey, their foraging rate will be lower than it would be at *X*_opt_. Because predators within food webs often consume more than one prey at a time, the interaction is often controlled by a multispecies type II functional response (Smout et al., 2010).

Under these assumptions, the mean intake rate of a predator for a given prey *i*, *f_i_*, can be written as follows:

$$f_{i}\left(R_{i},\;C,\;x,\;\bar{x},\;X_{\text{opt}},\;\sigma^{2}\right)=\int_{‐\infty }^{+\infty }\frac{\alpha_{i}\left(x,\;X_{\text{opt}}\right)R_{i}C}{1+\sum_{j=1}^{n}\alpha_{j}\left(x,\;X_{\text{opt}}\right)\eta_{j}\left(x,\;X_{\text{opt}}\right)R_{j}}p\left(x,\;\bar{x},\;\sigma^{2}\right)dx,$$

where the functions α and η represent the attack rate and handling time of the predator, respectively, $p\left(x,\;\bar{x},\;\sigma^{2}\right)$ is the underlying distribution of trait *x*, with mean $\bar{x}$ and variance $\sigma^{2}$, and where *R_i_* is the density of the *i*th prey, and *C* is the predator density (Gibert & DeLong, 2017).

The square distance between the optimum and the realized predator trait value can be seen as a measure of phenotypic mismatch (i.e. mismatch = ${\left(x‐X_{\text{opt}}\right)}^{2}$), or maladaptation. The smaller the value of the mismatch, the larger the intake rate of the predator (Gibert & Brassil, 2014; Gibert & DeLong, 2017). Assuming that the phenotypic mismatch varies across prey items in the diet of the focal predator (i.e. the predator is not perfectly well adapted to each of its prey), it is possible to assess how variation in the underlying trait variance, $\sigma^{2}$, changes the number and strength of all interactions.

As total phenotypic variation increases, both the number and strength of interactions change (Figure 1). In particular, lower amounts of variation lead to fewer but stronger interactions (Figure 1a) while larger amounts of variation lead to more, but weaker interactions (Figure 1b). Extending this analysis to multiple traits is also possible, as is taking into consideration prey trait distribution.

Plastic variation in the form of G × E can also rewire food webs. For example, pinyon pine genotypes express differential susceptibility to a sap‐sucking herbivore, resulting in dramatic differences in plant morphology (Stone et al., 2018). This change in plant morphology results in stark differences in the similarity and species richness of associated arthropod communities among plant genotypes under moderate drought. These differences are less pronounced, but still clear, under extreme drought. This example suggests that G × E can lead to network rewiring, as drought leads to changes in the effect size of plant genotypes on the arthropod community. In addition, a major determinant of predator–prey interactions—body size—may respond to changes in environmental conditions through G × E, as standing variation in body size has been shown to be partially explained by G × E in the fruit fly *Drosophila melanogaster* (Lafuente et al., 2018) and humans (Sulc et al., 2020). This, in turn, could have important consequences for food webs, as body size is a strong determinant of trophic level and interaction strength (Riede et al., 2011; Schneider et al., 2012).

While it would a priori be difficult to tease apart plastic rewiring due to E versus G × E, these sources of variation can now be teased apart using genomic and statistical tools, making it easier to quantify them in natural populations (Box 1; Rudman et al., 2018; Sulc et al., 2020). Alternatively, or in combination with these tools, common garden experiments can be established across relevant climate gradients or be manipulated directly to tease apart the sources of phenotypic variance (Cooper et al., 2019; Faticov et al., 2019).

### 4.1 Eco‐evolutionary consequences

**FIGURE 3:**
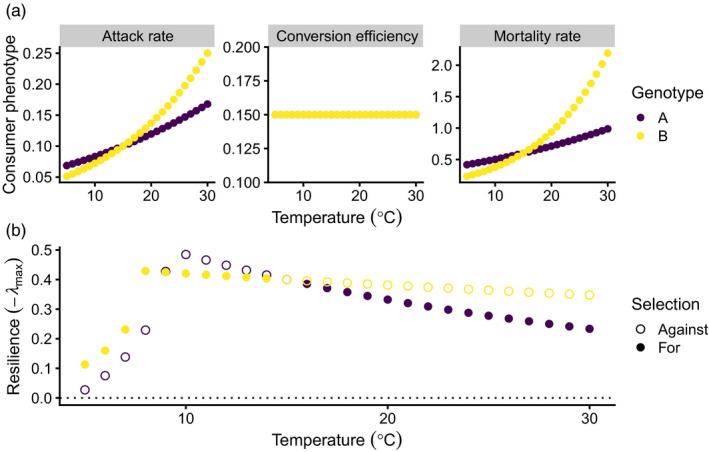
(a) Using a simple consumer–resource model (Box 3), we show how genotype‐by‐environment interactions (G × E) shape consumer phenotypes in response to temperature change. The different reaction norms for genotype A and B for attack rate and mortality rate represent a G × E, whereas the flat slope for conversion efficiency indicates no G, E or G × E. (b) These G × E effects on attack and mortality rates not only change which genotype is favoured by selection (solid versus open points) but also the resilience of the community to perturbations. Note that the community is always locally stable because $‐\lambda_{\text{max}}>0$ (i.e. above dotted line). Parameters and code to reproduce this figure are publicly available at https://mabarbour.github.io/foodweb‐theory/temperature‐GxE‐consumers.html

Shifts in E can influence both ecological and evolutionary dynamics, which also has consequences for food web structure. From a purely ecological perspective, changes in E can lead to shifts in transient dynamics for predator–prey interactions that sometimes result in instability, and sometimes result in increased stability, but almost always result in changes in equilibrium abundances (Gibert & Brassil, 2014; Gibert et al., 2015; Schreiber et al., 2011). Also, temperature is well known to plastically influence multiple ecological rates that ultimately determine ecological interactions: these effects can be both adaptive and plastic (Huey & Kingsolver, 1989, 1993; Schulte et al., 2011), depending on whether temperature exposure is acute or chronic (Schulte et al., 2011). For example, mortality rates often increase with temperature while growth rates respond plastically among ectotherms by increasing at first, then decreasing with temperature (Amarasekare, 2015; Amarasekare & Coutinho, 2014; Dell et al., 2011, 2014; Savage et al., 2004; Uszko et al., 2017). As a consequence, increasing temperatures and temperature fluctuations will affect E in growth, mortality and other ecological rates (Box 3), which will, in turn, influence selection acting on interacting species (Box 3). Changes in E can therefore influence the adaptive landscape and mediate the eco‐evolutionary dynamics of interacting species in changing environments (Figure 3). Current eco‐evolutionary models, however, rarely account for a scenario in which E may change over time (but see Fischer et al. 2014), even though very large levels of phenotypic variation and low levels of heritability have been considered to account for fixed levels of E (McPeek, 2017).

If the expression of genetic variation changes with the environment (G × E), eco‐evolutionary dynamics within food webs will also be affected. For example, if selection is stronger in more variable environments, then forms of G × E that increase the expression of G are expected to drive rapid evolution (Wood & Brodie III, 2015). In contrast, if the expression of G decreases, a strong evolutionary response is unlikely, even if selection is strong (Wood & Brodie III, 2015). Despite evidence for G × E in shaping phenotypic variation in ecologically important traits (Lafuente et al., 2018; Sulc et al., 2020), the role of G × E in shaping eco‐evolutionary dynamics remains unknown. To understand these effects, we use a simple consumer–resource model and tools from adaptive dynamics to explore how changes in temperature alter the dynamics of simultaneous trait evolution and changes in abundances (Box 3). When consumers exhibit G × E in phenotypes that determine consumer–resource interactions (‘attack rate’ and ‘mortality rate’ panels, Figure 3a), changes in temperature alter which genotype is favoured by natural selection (solid points in Figure 3b). This evolutionary change alters the resilience of the community to perturbations (Figure 3b), potentially leading to secondary species extinctions (Dunne et al., 2002; McCann, 2000). In particular, evolution may result in more resilient community dynamics under certain temperature regimes but lead to less resilient communities under different temperature conditions (Figure 3b). This nonlinear relationship between evolution and community resilience is likely quite common, given that consumer phenotypes have a nonlinear relationship with resilience (Rip & McCann, 2011). Interestingly, we also see that the effect of G on resilience increases with warming, as indicated by the greater difference between solid and open points in Figure 3b. This suggests that climate change may magnify the importance of evolutionary dynamics for community stability. We note that we currently do not know what G × E looks like in the context of temperature change for many of these parameters of this simple model, so our model should be taken as a proof‐of‐concept. Still, even this simple model illustrates the diverse ways in which G × E effects could rewire food webs over time (Box 3).

BOX 3 **Incorporating G × E effects in an eco‐evolutionary model**

Consider a simple model of consumer–resource interactions, written in terms of per‐capita population growth, a common metric of absolute fitness (Amarasekare & Savage, 2012; Lande, 1976), of the consumer (*C*) and resource (*R*):

$\frac{1}{R}\frac{dR}{dt}=r‐\frac{r}{K}R‐aC,$

$\frac{1}{C}\frac{dC}{dt}=eaR‐m,$

where *r* corresponds to the intrinsic growth rate of the resource, *K* is its carrying capacity, *a* is the consumer's per‐capita attack rate, *e* is the conversion efficiency of resources into new consumers and *m* is the per‐capita mortality rate of the consumer. This is a starting point for most consumer–resource models because of its simplicity but general behaviour (McCann, 2011).

Most studies addressing the impacts of climate warming on ecological interactions incorporate well‐known temperature dependencies of the above parameters into their models (Amarasekare, 2015; Gilbert et al., 2014; Sentis et al., 2017; Uszko et al., 2017). For example, mortality rates often increase with temperature while intrinsic growth rates increase then decrease with temperature (Amarasekare & Savage, 2012). We argue that such models depict the average plastic response among genotypes of the traits that control the interaction parameters to changes in temperature, or the effects of a change in E.

To understand how genotype‐by‐environment interactions (G × E) might alter ecological dynamics, let us consider two different genotypes of the consumer (A and B). These genotypes have the same ‘initial’ phenotype at 15℃. Then, changes in the thermal sensitivity of the parameter (i.e. the slope of the temperature relationship with the parameter) would effectively represent a G × E effect. This makes it possible to visualize G effects (comparing the genotypes at 15℃), E effects (mean phenotype change with temperature) and G × E effects (different slopes of each genotype, Figure 3a). Moreover, using standard tools from eco‐evolutionary theory (Otto & Day, 2007), we can assess how G, E and G × E affect ecological dynamics, as evolutionary and plastic change unfolds across the temperature gradient (Figure 3b).

In this example, we only model the effect of G × E in the consumer's phenotype for simplicity but also account for E in the resource. This model suggests that the thermal sensitivity of genotype A’s (purple) attack and mortality rate is less than genotype B (yellow). This difference in thermal sensitivity alters not only which genotype is favoured by selection (solid vs. open points) but also the resilience of the consumer–resource system to perturbations (i.e. return time to equilibrium).

## 5 ASYMMETRY IN RESPONSES AMONG INTERACTING SPECIES

### 5.1 Asymmetries lead to rewiring

While eco‐evolutionary models routinely account for changes in mean phenotypes (Ellner et al., 2011; Jones et al., 2009; McPeek, 2017; Van Velzen & Gaedke, 2017; Yeakel et al., 2018), interacting species are unlikely to respond to climate change in the same way, leading to what have been dubbed ‘asymmetric temperature responses’ (Barton & Schmitz, 2009; Kingsolver, 2009; O'Connor, 2009; Rall et al., 2010). Changes in mean phenotypes are well known to affect ecological dynamics as they unfold, but the potential for asymmetric phenotypic change between interacting species has received little attention. We hypothesize that mean changes in phenotypes are likely to lead to large network‐level rewiring only when they are asymmetric.

**FIGURE 4:**
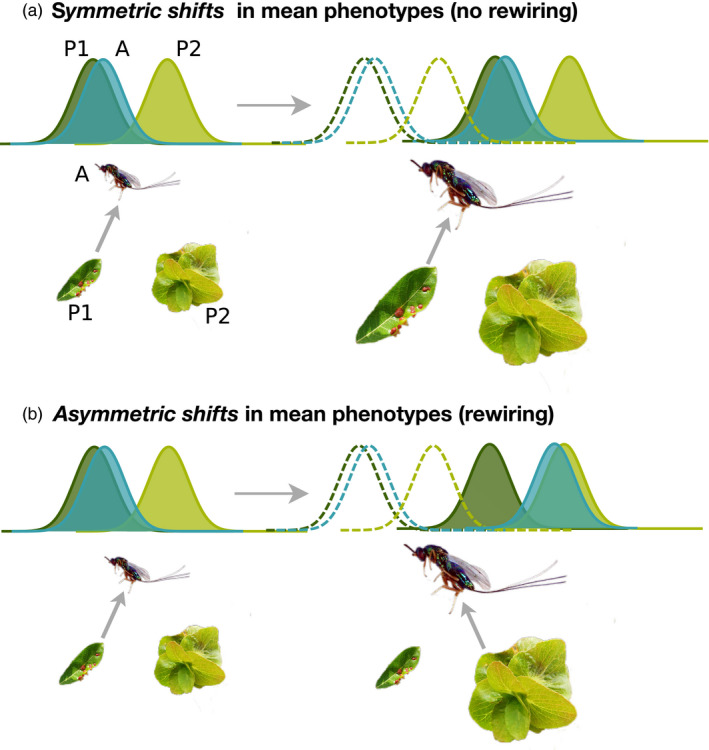
(a) Symmetric shifts in mean phenotypes maintain body size ratios, and existing levels of phenotype matching between the parasitic wasp and gall making insects, therefore maintaining existing interactions. (b) Asymmetric shifts in mean phenotypes, on the other hand, can lead to new interactions through changes in phenotypic matching

Asymmetric plastic responses to temperature have been shown to influence population and community‐level dynamics in ecological models (Dell et al., 2014; Grady et al., 2019; O'Connor et al., 2011). In eco‐evolutionary models, genetic variation (G) is often assumed to be the same among interacting species, creating an equal potential for evolutionary change (but see Cortez, 2016, 2018). Therefore, asymmetric evolutionary responses can only arise in these models due to differences in selection or generation time. Asymmetry in G, while likely to be the norm, has not received as much attention in the literature as symmetric responses among interacting species have (but see Cortez, 2018). Yet, symmetric change in mean phenotypes is unlikely to significantly alter the phenotypic matching that ultimately determines the outcome of ecological interactions. Symmetric phenotypic change is therefore unlikely to result in large‐scale rewiring of ecological networks, for example, through the maintenance of predator–prey body size ratios within food webs (Figure 4a). On the other hand, asymmetric changes in phenotypes in a focal species, adaptive, plastic or otherwise, may increase phenotypic matching with some species in the network, while increasing levels of mismatch with other species, effectively rewiring the network (Figure 4b).

### 5.2 Species role within food webs mediates genetic and plastic responses

**FIGURE 5:**
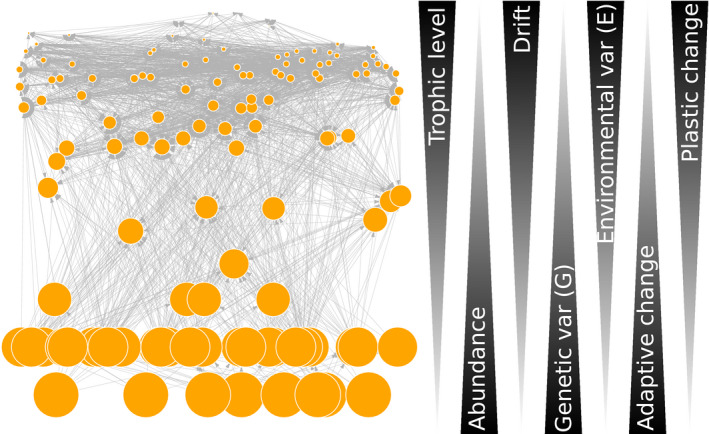
Orange nodes indicate species and grey arrows, feeding interactions (Carpinteria food web, Lafferty et al., 2008). Node size represents species abundance (putatively), and trophic level increases from the bottom to the top of the food web. We hypothesize that as abundance decreases with trophic level, increasing levels of genetic drift are likely responsible for lower G at higher levels, but comparably larger E. As a consequence, adaptive evolutionary change is more likely to occur at lower trophic levels while plastic responses are more likely to occur at higher trophic levels, leading to differences in the way organisms cope with a rapidly changing climate across trophic levels

Systematic asymmetries in genetic (G) and plastic (E and G × E) trait variation are likely across trophic levels (Figure 5). For example, species that occupy higher trophic levels are likely to have lower levels of genetic variation due to smaller population sizes (Frankham, 1996). As a consequence, the capacity to adapt to climate change is likely to be lower at higher trophic levels (Scheffers et al., 2016). Indeed, the distribution of genetic diversity among animal species may be explained by traits related to parental investment, where long‐lived or low‐fecundity species (that usually occupy higher trophic levels) are genetically less diverse than short‐lived or highly fecund ones (which are typically found closer to the bottom of the food web) (Romiguier et al., 2014). For example, the bottom of pond food webs is dominated by short‐lived unicellular organisms (algae and bacteria) consumed by slightly larger but also short‐lived unicellular or multicellular organisms (protists, rotifers). In these systems, eco‐evolutionary dynamics have been shown to be common, as bacteria and algae evolve rapidly to predation by zooplankton (Becks et al., 2012; Frickel et al., 2016, 2017; Yoshida et al., 2003). In contrast, the longer‐lived animals that occupy higher trophic levels are likely to exhibit more phenotypic plasticity (Figure 5). Interestingly, plants and modular animals appear to be much better equipped with plasticity mechanisms than unitary animals (Borges, 2008), at least in terms of morphological or physiological plasticity. The story is even more complex when considering behavioural flexibility (another form of phenotypic plasticity), which likely increases with trophic level (Edmunds et al., 2016). Indeed, species behaviour has been shown to mediate food web temperature responses through the landscape of fear (Barton & Schmitz, 2009).

A major consequence of these hypotheses is that we expect genetic rewiring to be much more common at the bottom of the food web than at the top and plastic rewiring to be more common at the top than at the bottom, as the climate changes. While this is the general expectation for many food webs, where body size plays an important role in mediating who eats whom, there are also clear exceptions based on interacting species’ life‐history traits. For example, we would expect genetic rewiring to be more common at the top of mammal host–parasite networks where the parasites (at the top of the web) have much faster life‐history strategies. In insect host–parasitoid networks, however, the potential for genetic and plastic rewiring may be equally prevalent, as life‐history strategies are more similar between trophic levels. Regardless of the type of food web, we hypothesize that both plastic and genetic rewiring are likely to occur within the same network, perhaps simultaneously, but at different trophic levels, thus altering the way organisms that play different structural roles may respond to a rapidly changing environment.

## 6 CONCLUSIONS

Here, we present a synthesis of concepts from food web theory and quantitative genetics to provide hypotheses as to how shifts in environmental conditions may affect the structure and dynamics of food webs (Table 1). In particular, we hypothesize that environmentally induced increases in genetic variation (G) should lead to more, weaker interactions, whereas decreases in G should lead to fewer, but stronger interactions in food webs (Table 1a). We hypothesize that climate change should typically lead to increases in phenotypic plasticity (E and G × E), resulting in more numerous, but weaker interactions (Table 1b). We hypothesize that symmetric plastic or genetic shifts in mean phenotypes are unlikely to rewire food webs (Table 1c), while asymmetric responses will result in rewiring (Table 1d). We also hypothesize that changes in genetic and plastic trait variation are likely to mediate eco‐evolutionary responses, which will, in turn, affect the way food webs respond to climate change. Last, we hypothesize the existence of systematic differences across trophic levels that will make them more or less susceptible to plastic and genetic rewiring as the climate changes. We hope this synthesis will open new fields and modes of inquiry at the interface between ecology, evolution and climate change biology, and provide a much‐needed lens through which to understand how climate‐related effects may be mediated by individual traits and their genetic makeup within populations, communities and ecosystems.

**TABLE 1:** Hypotheses for how climate change will genetically and plasticly rewire food webs and alter eco‐evolutionary dynamics

Hypothesis	Process	Predictions				
		Direction of change	Food web response	Evolutionary response	Relative importance	Pace
Changes in phenotypic variation						
(a) Genetic (G) rewiring	Gene flow or disruptive selection	Increase in G	More but weaker interactions	Increase in importance	Lower trophic level, short‐lived, high‐fecundity species (trees are an exception)	Medium
	Genetic drift; directional or stabilizing selection	Decrease in G	Fewer but stronger interactions	Decrease in importance		Slow
(b) Plastic (E and G × E) rewiring	Novel environmental heterogeneity	Increase in E and G × E	More but weaker interactions	Increase or decrease	Higher trophic level, long‐lived, low‐fecundity species	Fast
Changes in mean phenotypes						
(c) Symmetric phenotypic change	Equal selection, G, E or G × E	No change in phenotypic mismatch	No change	No change		Fast, Medium, Slow
(d) Asymmetric phenotypic change	Unequal selection, G, E or G × E	Mismatch increases with some species, decreases with others	Rewiring	Increase in importance	Interacting species at different trophic levels, life‐history strategies	Fast, Medium, Slow

## AUTHORS' CONTRIBUTIONS

M.A.B. and J.P.G. conceived the ideas in this manuscript, did the literature review and wrote the paper; M.A.B. did the G × E modelling presented in Box 3 while J.P.G. did the modelling presented in Box 2.

## Data Availability

Annotated code is publicly available on GitHub (https://github.com/mabarbour/foodweb‐theory) and we also provide a more user‐friendly website to view the code (https://mabarbour.github.io/foodweb‐theory/temperature‐GxE‐consumers.html). This code has been archived with Zenodo (https://doi.org/10.5281/zenodo.4774839) (Barbour, 2021). No data was used.
